# Hygiene-based measures for the prevention of cytomegalovirus infection in pregnant women: a systematic review

**DOI:** 10.1186/s12884-024-06367-5

**Published:** 2024-02-29

**Authors:** María F. Rodríguez-Muñoz, Clara Martín-Martín, Katina Kovacheva, Maria Eugenia Olivares, Nuria Izquierdo, Pilar Pérez-Romero, Estéfani García-Ríos

**Affiliations:** 1 Faculty of Psychology, Universidad Nacional de Educación a Distancia, (UNED), Madrid, Spain; 2 National Centre for Microbiology, Instituto de Salud Carlos III (ISCIII), Carretera Majadahonda - Pozuelo km. 2, Majadahonda, Madrid 28220 Spain; 3 Department of Gynecology and Obstetrics, Hospital Clínico San Carlos, Madrid, Spain; 4 Department of Biological Sciences, University of Notre Dame, Notre Dame, IN 46556 USA; 5 Department of Food Biotechnology, Instituto de Agroquimica y Tecnologia de los Alimentos (IATA), CSIC, Agustín Escardino 7, Paterna, Valencia 46980 Spain

**Keywords:** Systematic review, Cytomegalovirus, Pregnancy, Counselling, Seroconversion

## Abstract

**Background:**

Human Cytomegalovirus (HCMV) is the most frequent congenital infection worldwide causing important sequelae. However, no vaccine or antiviral treatments are currently available, thus interventions are restricted to behavioral measures. The aim of this systematic review was to assess evidence from available intervention studies using hygiene-based measures to prevent HCMV infection during pregnancy.

**Methods:**

Studies published from 1972 to 2023 were searched in Medline, PsycInfo, and Clinical Trials (PROSPERO, CRD42022344840) according to the Preferred Reporting Items for Systematic Reviews and Meta-Analyses (PRISMA) guidelines. Methodological quality was assessed by two authors, using ROBE-2 and MINORS.

**Results:**

After reviewing 6 selected articles, the outcome analysis suggested that implementation of hygiene-based interventions during pregnancy prevent, to some extent, the acquisition of congenital HCMV.

**Conclusions:**

However, these conclusions are based on limited and low-quality evidence available from few studies using this type of intervention in clinical practice. Thus, it would be necessary to perform effective and homogeneous intervention studies using hygiene-based measures, evaluated in high-quality randomized controlled trials (RCTs).

**Supplementary Information:**

The online version contains supplementary material available at 10.1186/s12884-024-06367-5.

## Background

Human Cytomegalovirus (HCMV) belongs to the *Herpesviridae* family (HHV-5), and it is the most frequent congenital infection worldwide (0.5 to 5%), causing important sequelae such as sensorineural hearing loss (SNHL) and neurodevelopmental disabilities in newborns [1–3]. In reproductive age women, HCMV seroprevalence varies based on socioeconomic factors [4]. While in developed countries in Europe and North America, seroprevalence ranges from 40 to 83% [5, 6], in developing countries from Asia, Africa, and Latin America, seroprevalence can reach 100% [7]. The prevalence of congenital infection (cHCMV) is therefore associated with seroprevalence among pregnant women, with a higher seroprevalence correlating with ahigher prevalence of cHCMV infection [8]. In developed countries where up to 50% of childbearing age women are seronegative, cHCMV infection usually occurs due to frequent contact with small children (< 3 years of age) [9, 10].

During primary infection, viral shedding can occur through saliva, urine, breast milk, semen, and blood [11]. A primary infection takes place when an individual with no previous HCMV infection (HCMV seronegative individuals) are infected through contact with a HCMV infected individual, triggering a broad immune response and establishing lifelong latency [12]. Seropositive individuals can develop new HCMV infection episodes from viral reactivation of latent infection or through re-infection with different viral strains [12–14]. During pregnancy, HCMV transmission to the fetus can occur both from mothers with primary infection (seronegative women) and from mothers with reactivated latent infection where hormonal changes associated with pregnancy and lactation may stimulate the reactivation [15, 16].

The greatest risk of vertical transmission is associated with primary infection ranging from 30 to 35%, compared to 1.1 to 1.7% for non-primary infections [17]. However, it is crucial to consider the likelihood of acquiring an infection. The risk appears to be relatively low for seronegative women and relatively high for seropositive women [18]. This observation is supported by earlier studies that modelled the force of infection, estimating a higher incidence in highly seropositive populations [19, 20], likely due to environmental and behavioural differences. Some studies indicate that the risk of re-infection among seropositive women surpasses the combined risks of both acquisition and maternal-to-fetal transmission among seronegative women [21]. Recent serological studies examining strain-specific HCMV antibody responses have revealed that maternal re-infection with a new strain is a significant factor in congenital infection among seropositive women, with re-infections occurring in 8% of seroimmune pregnancies [22].

Other authors have reported a much greater proportion of symptomatic cHCMV linked to reinfection during pregnancy [23, 24]. Up to 10% of neonates with cHCMV infection are symptomatic and develop different sequelae. cHCMV is the leading cause of sensorineural hearing loss (SNHL) and neurodevelopmental delay, with a large number of symptomatic children presenting some degree of psychomotor and cognitive disabilities, and with visual impairment in up to 50% of infants [25–28]. Likewise, several studies have demonstrated that the risk of long-term neurodevelopmental sequelae, specifically hearing loss, is comparable in infants born to women with primary HCMV and those with non-primary HCMV infections during pregnancy [29–31]. The burden of disease related to cHCMV infection is notable, and as a consequence, infants often require special care related to therapeutic and educational needs [3, 32].

Currently, there is no licensed vaccine to prevent HCMV infection and no approved treatments to prevent viral transmission from mother to fetus [19, 33–38]. Neonates with symptomatic infection can be treated with ganciclovir and/or valganciclovir for 6 months [28]. Although this therapy has been shown to modestly reduce the incidence of hearing loss [39], follow-up duration is limited [40] and further research may be needed. Furthermore, paediatric administration of ganciclovir or valganciclovir is associated with neutropenia, and monitorization of neutrophil counts is recommended in treated children [39, 41]. Thus, to reduce transmission from the mother to the fetus and consequently reduce the global burden of HCMV disease in this population, current interventions are restricted to behavioural changes to promote prevention measures. The literature has highlighted three types of prevention measures: universal (targeting the general population, not directed to a specific risk group), selective (targeting individuals at higher-than-average risk for HCMV infection) and indicated (individuals who are identified as having an increased vulnerability for HCMV infection). In our case, the universal group would be pregnant women; the selective group would be seropositive pregnant women for HCMV infection, and the indicated group those pregnant seronegative to HCMV and, thus they are at higher risk of transmission. Selective and indicated prevention strategies might involve more intensive interventions. To identify effective intervention studies, the studies should have described which types of hygiene prevention measures are adequate, determined the preventive effect of the interventions to avoid infection, and generated high level evidence.

To the best of our knowledge, only two review manuscripts describing HCMV preventive interventions have been conducted to date in the general population [42, 43]. However, neither of the systematic reviews established which type of intervention is most appropriate for preventing HCMV infection. The first review focused exclusively on trials published before 2004 [42] while the second focused on trials published before 2019 [43]. Although both studies included content on behavior modifications, none of them used the Psyinfo database specialized in this field.

Thus, the existing evidence on preventive interventions for HCMV infection in the perinatal period remain inconclusive. For this reason, the aim of this review is to collate evidence relating hygienic measurements acquisition and counselling during pregnancy in order to reduce cHCMV infection.

## Methods

### Search procedures and eligibly criteria

This systematic review of published studies was conducted following the Preferred Reporting Items for Systematic reviews and Meta-Analysis (PRISMA) statement [20, 44]. A systematic review between 1972 and 2023, written in English (due to resource limits), assessing preventive intervention for HCMV infection was carried out. Database search was conducted in September 2023 by two authors (MFR and EGR) independently.

A protocol was elaborated to implement different steps underlying this systematic review and was registered on PROSPERO, the International Prospective Register of Systematic Reviews (ID CRD42022344840). No deviations from the protocol have occurred.

A total of three electronic databases were searched: MEDLINE, PsycINFO. In addition, CLINICAL TRIALS.gov was used to identify unpublished studies or studies still ongoing. The following search terms were combined: (“cytomegalovirus” OR “CMV” OR “CCMV” OR “HCMV” OR “Human betaherpesvirus 5” OR “cytomegalovirus infection*” OR “CMV infection*” OR “cCMV infection*” OR “HCMV infection*” OR “Human betaherpesvirus 5” infection*” OR “congenital cytomegalovirus infection*” OR “congenital infection*” OR “congenital CMV” OR “congenital HCMV” ) AND (“prenatal*” OR “pre-natal*” OR “pre natal*” OR “antenatal* OR “ante-natal*” OR “antepartum” OR “ante-partum” OR “pregnancy” OR “pregnant*” OR “mother*” OR “childbearing” OR “woman” OR “women”) AND (“prenatal education*” OR “antenatal education*” OR “birth preparation*” OR “prenatal class*” OR “antenatal class*” OR “health education*” OR “health promotion*” OR “counselling*” OR “hygiene*” OR “hand wash*” OR “wash hand*” OR “program” OR prevention” OR “control” OR “hygiene-based” OR “control” OR “hygiene education” OR “behavioral intervention” OR “Vertical prevention” ).

### Inclusion and exclusion criteria

Analysis of the articles was performed based on previously established inclusion and exclusion criteria and the availability of the full text in English. Randomized controlled trial (RCT) and non-RCT about the effectiveness of HCMV acquisition were eligible for inclusion in the systematic review. The review included articles studying adult pregnant women or attempting pregnancy to whom preventive intervention based on hygiene education and control groups receiving standard treatment or information. The primary outcome of the review was the measurement of seroconversion rates and the secondary was the adherence of pregnant women to the intervention.

Search results were exported to an Excel file and duplicate manuscripts were removed. Two authors (MFR and EGR) independently screened titles and abstracts for eligibility and the full text of the potentially eligible articles were screened. Studies were excluded if they did not evaluate the effectiveness of preventive intervention for HCMV, or did not include psychological or biological outcomes. Any disagreement was resolved by discussion.

### Data extraction

Two reviewers (MFR and EGR) independently extracted data from each included study and checked for accuracy using a data extraction excel spreadsheet. The following data were extracted: aim of study, author, year of publication, country of study, time of study, study design, inclusion/exclusion criteria, characteristics of cohort, description of interventions’ characteristics (data were collected as narrative results) – type (information or counselling); delivery format (face-to-face, written, video, individual, group, online); time of intervention (pregnancy or attempting pregnancy); duration of intervention (range); number of sessions (range); follow-up duration; providers; outcome measures and main findings. Summary tables were made to create the extracted information in an organized presentation. Excluded studies and reason for the exclusion has been included in Supplementary material (Table S1).

### Risk of bias assessment

Methodological quality of the included studies was independently assessed by two reviewers (MFR and EGR) using ROB-2 [45], a tool developed for assessing the quality of randomized health care interventions and MINOR [46] for non/randomized intervention [47]. ROB-2 evaluates five domains of research validity and bias: randomization process, deviations from intended interventions, missing outcome data, outcome measurement and selection of the reported results. Studies were evaluated as either low, some concerns or high risk of bias for each domain. MINORS contained 12 items, the first eight being specifically for non-comparative studies. The items are scored 0 (not reported), 1 (reported but inadequate) or 2 (reported and adequate). The global ideal score being 16 for non-comparative studies and 24 for comparative studies.

Risk of bias was categorized as low or high. Disagreements on quality rating were discussed and a consensus was reached.

## Results

### Identification of studies

**Fig. 1:**
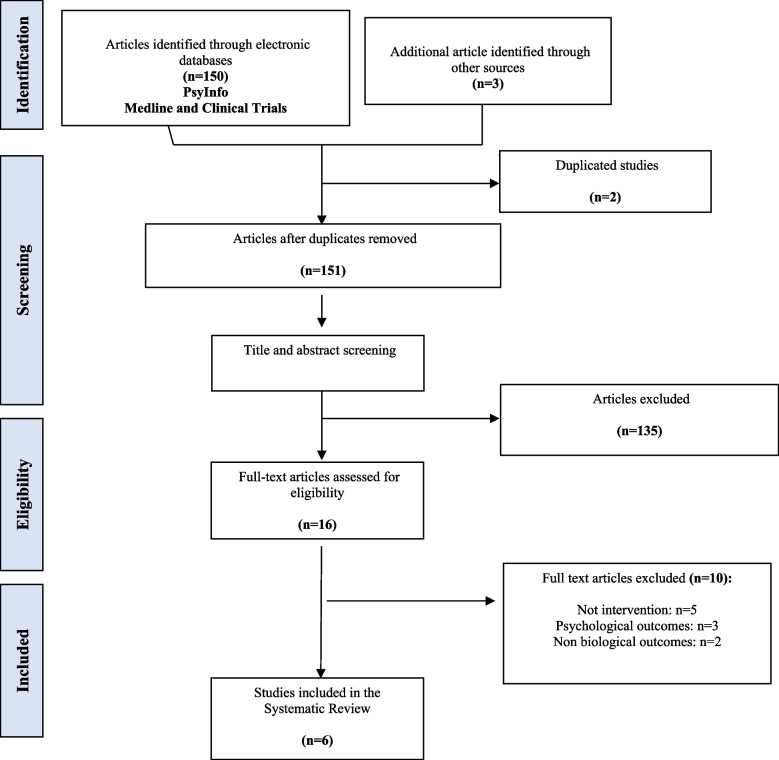
PRISMA flow-chart

Search results are summarized in the PRISMA flowchart (Fig. 1). The initial search identified a total of 150 references and 3 additional records were collected based on experts in the field. After removing duplicate references, the title and abstract, a total of 145 references were screened (first screening) and a total 135 studies were excluded. A full-text review was performed for the remaining 16 references (second screening) and based on eligibility assessment 10 records were excluded (exclusion reasons are presented in Table S1) and six articles were included in the systematic review. The methodological quality and bias risk of the included studies are shown in Table 1. All four non-RCT papers [48–51] were classified as critically low-quality using MINORS, mainly due to unbiased assessment of the study, for lacking a follow-up period or a prospective calculation of the sample size. In contrast, the two RCT papers [52, 53] were evaluated with a high quality based on ROBE-2 criteria.

**Table 1:** Risk of bias of RCT and non-RCT studies included in the systematic review

Study	Type of study	Domain 1			Domain 2			Domain 3				Domain 4			Domain 5			Quality
Adler et al., (2004) [52]	RCT	Low			Low			Low				Low			Low			High ^a^
Calvert et al., (2021) [53]	RCT	High			Low			Low				Low			Low			High ^a^
		Item 1	Item 2	Item 3		Item 4	Item 5		Item 6	Item 7	Item 8		Item 9	Item 10		Item 11	Item 12	
Valoup-Fellous et al., (2009)	Non-RCT	2	1	0		2	0		2	2	0		NA	NA		NA	1	Low ^b^
Picone et al., (2009) [50]	Non-RCT	2	2	0		2	0		2	2	0		NA	NA		NA	2	Low ^b^
Reichman et al., (2014) [48]	Non-RCT	2	2	0		0	0		2	2	0		NA	NA		NA	2	Low ^b^
Revello et al., (2015) [49]	Non-RCT	2	2	0		2	0		2	1	1		2	2		1	2	Low ^b^

*0 *not reported, *1 *reported but inadequate, *2 *reported and adequate, *NA *Not applicable. ^a^ – quality assessment by RoB2 (Sterne et al., 2019); ^b^ – quality assessment by MINORS tool (Slim et al., 2003)

### Study characteristics

Characteristics of the six included papers are shown in Table 2. All the studies were published between 2004 and 2021 reporting the findings from a total number of 10,197 participants (i.e., pregnant women or women who attempt to be pregnant). The studies were carried out in United States [52]; Italy [49]; Israel [48]; United Kingdom [53] and France [50, 51]. Sample size ranged from 103 to 5173 women with a median of 545 and a mean of 1699.5.

**Table 2:** Characteristics of the included studies

Authors		Aim	Setting	Study characteristics	Intervention’ characteristics	Control group	Outcomes
Adler et al. (2004) [52]	To determine if protective behavior prevents child-to-mother transmission of HCMV during pregnancy		Seronegative mothers with a child < 36 months of age Childcare centres in Central, Northern, and Eastern Virginia, (USA), from 1999 through 2001	*N* = 166 (IG: *n* = 92; Full IG: *n* = 23; CG: *n* = 51) Cluster RCT study Not reported funding	*Intervention (serologic status known but child’s shedding unknown)*: written, oral and video information + adherence visits. Liquid soap and latex gloves were provided *Full intervention (serologic status and child’s shedding known)*: written, oral and video + adherence visits. Liquid soap and latex gloves were provided	The women received basic information about HCMV. They were not aware of their serologic status or whether their child was shedding HCMV	a) Maternal HCMV seroconversion b) Measures of adherence: percentage of times per biweekly that mothers performed or avoided a specific behaviour was estimated. The number of gloves remaining was monitored, the amount of soap remaining was weighed and the number of diapers remaining was counted
Picone et al. (2009) [50]	To evaluate the frequency of pregnant women agreeing to HCMV serologic screening after information of the consequences of HCMV infection		Pregnant women received information of HCMV infection during two years between January 2005 and December 2006. Service de Gynécologie-Obstétrique, Hôpital Antoine Béclère, Clamart, France	*N* = 3665 Prospective cohort study No funding	*HCMV-seronegative*: detailed oral and written hygiene information + second test at 36WG + systematic ultrasound examinations at 12, 22, 32 WB *HCMV-seropositive*: monthly fetal ultrasound examination *Seroconversion between 12–36 WB: *ultra- sound examination + transvaginal evaluation of fetal brain	NA	a) Rate of HCMV seropositive and seronegative women b) Rate of women consent for screening c) Rate of primary infection d) Rate of seroconversion e) Number of HCMV-infected newborns
Vauloup-Fellous et al. (2009) [51]Vauloup-Fellou	To evaluate the impact of a prenatal HCMV infection screening and counselling policy		Pregnant women who had their first medical visit to an obstetric department between January 2005 and December 2007 Service de Gynécologie-Obstétrique, Setting: Hôpital Antoine Béclère, Clamart, France	*N* = 5173 Prospective cohort study Not reported funding	*HCMV-seronegative*: detailed oral and written hygiene information + second test at 36WG + contact telephone number for further information + systematic ultrasound examinations at 12, 22, 32 WB *HCMV-seropositive*: monthly foetal ultrasound examination *Seroconversion between 12–36 WB: *ultra- sound examination + transvaginal evaluation of the foetal brain	NA	a) Rate of women agreeing for screening b) Rate of primary infection c) Rate of seroconversion d) Number of HCMV-infected newborns
Reichman et al. (2014) [48]	To assess the effect of counselling preconception		Women who planned pregnancy and were referred to a fertility clinic. Outpatient fertility clinic at Shaare Zedek Medical Centre, Jerusalem, Israel over a 28-month period	*N* = 444 Retrospective Cohort study Not reported funding	HCMV testing and counselling at preconception *Seronegative women*: advised to adopt behaviours and follow-up evaluation of their HCMV immunity status every 3–4 months *Women primary infection*: advised to postpone pregnancy for 6–9 months *Women remote infection*: continued with infertility treatment	NA	Preconception screening for HCMV *Seronegative*: IgG (−)/IgM (−) *Seropositive*: IgG (+)/IgM (−) *Seroconversion*: (IgG (+) high avidity/IgM (+) (past primary infection or reactivation) or IgG (+) low avidity/IgM (+) (primary infection)
Revello et al. (2015) [49]	To investigate the effectiveness of information and hygiene recommendations for prevention of HCMV infection and to assess acceptance of and adherence to hygiene		Pregnant women at high risk for primary HCMV infection (seronegative) Two participating centres were involved, one in Pavia and one in Turin (Italy)	*N* = 646 (IG: *n* = 331; CG: *n* = 315) Interventional and observational controlled study Funded by Fondazione Carlo Denegri, Torino, Italy	The study time covered the period from 11–12 weeks of gestation to delivery Seronegative women or unknown immune status received written and oral information + reinforcement + adherence questionnaire	Women who were not tested or informed about HCMV during pregnancy but who have undergone foetal aneuploidy screening at 11–12 WG. At the time of delivery, both seronegative and susceptible women were informed of the potential risks associated with HCMV susceptibility in future pregnancies	HCMV seroconversion screening and self-report of adherence to hygiene recommendations
Calvert et al. (2021) [53]	To examine the efficacy of an antenatal digital intervention to reduce the risk of HCMV acquisition in pregnancy		Women in their first trimester of pregnancy who were attending antenatal clinics between September 2018 and September 2019 Teaching hospital in an ethnically diverse area of South-West London (UK)	*N* = 103 (IG: *n* = 51; CG: *n* = 52) RCT study Not reported funding	Women watched educational film about HCMV (prevalence and routes of transmission; families of affected children and advice to minimise the risk of HCMV infection) + baseline and 34-week questionnaire	Women viewed a series of slides about influenza vaccination in pregnancy + Baseline and 34-week questionnaire	Differences in knowledge about HCMV, perceived severity, susceptibility and HCMV risk reducing behaviour of pregnant women Anxiety and depression scores Seroconversion

*HCMV *Human Cytomegalovirus, *RCT *Randomized Controlled Trial, *IG *Intervention Group, *CG *Control group, *WG *Week´s Gestation, *NA *Not Available, *IgG *Immunoglobulin G, *IgM *Im

As previously stated, two of the studies were RCTs [52, 53] while the remaining four paper were a retrospective cohort study [48]; an observational controlled study [49] and two prospective cohort studies [50, 51]. Four studies focused exclusively on pregnant women [49–51, 53]. One study on women who were planning to be pregnant [48] and the last one included both, pregnant women and women who were planning to be pregnant [52].

Regarding the type of prevention approach used, five studies included selective prevention in seronegative women [49–53] and finally one paper focused on universal prevention [48].

Regarding the type of personnel providing the interventions, in four of the studies the interventions were mainly provided by health professionals (nurses, midwives, gynecologists) while in two of the studies this information was not reported [49, 53].

## Characteristics of the interventions

All six studies evaluated the effectiveness of the preventive intervention on the reduction of HCMV acquisition (seroconversion). Most of the papers focused on informing patients of preventive measures [49–53]. However, some of the studies in addition to informing patients of preventive measures, included other parameters such as adherence to follow up visits [52], patient follow up by telephone calls [51] and patient reinforcement [49]. Only one of the selected studies included psychological support through counseling [48].

Table 3 shows a summary of the main results. In more detail, Adler et al., (2004) analyzed 166 seronegative women with a child below 36 months of age. In this work, participants were randomly assigned to either the control group (intervention) or the intervention group (full intervention). In the control group, women received basic information about HCMV infection but they were not aware of their serological status or whether the child was shedding HCMV or not. On the contrary, women assigned to the intervention group received the same information and indications as in the control group but additionally they were aware of their serological status and whether their child was shedding HCMV or not, and their implications. In addition, home visits were carried out every 3 months in order to assess adherence to the measures of both groups.

**Table 3:** Effectiveness of preventive interventions

Authors	Main findings
Adler et al. **(**2004) [52]	*Recruitment and attrition*: 42 out of 234 enrolled women were excluded as HCMV seropositive at enrolment and 26 failed to provide the follow-up specimens. *Treatment adherence*: Although there was no association between adherence measures and infection rates, it seems that intervention was more successful in pregnant women than in non-pregnant women as they were more committed to behavior modification. For example, women who were pregnant at the time of registration reported kissing their child on the lips half as often as women who were not pregnant. In addition, these objective and subjective measures may not have reflected actual practice. Control group: 7.8% seroconverted; Intervention group: 7.8% (*P* = 1). There was a significant association between maternal infection and children excreting HCMV at any time and attempted pregnancy at enrolment. In addition, not being pregnant at enrolment significantly increased risk of acquisition (*P* < 0.0001). Pregnant: 5.9% Attempting pregnancy: 41.7%; *P* = 0.008 Prenatal counselling for HCMV infection in pregnant women at the first obstetric visit may reduce the risk of HCMV infection. Behavioral intervention as preventive measure for seropositive pregnant women with young children in day care may have broad public health impact.
Picone et al. **(**2009) [50]	*Recruitment and attrition*: Of the 4,287 pregnant women at baseline, 495 were already HCMV seropositive before the first visit to the center. Of the remaining 3,792, 127 refused screening. The infection rate between 12–36 WG was significantly lower (0.01% pregnant woman-week) than the infection rate before 12 WG (0.04% pregnant woman-week) (mid *P* = 0.02, 95% CI [1.07–13.6]). HCMV-seroconversion: 0–12 WG: 0.46% of pregnant women, 12–36 WG: 0.26% of pregnant women (CI: 1.07–13.6; mid *P* = 0.02, 95%). Of the 5 women who seroconverted between 12–36 WG, 2 of the newborns were infected but asymptomatic. Among the nine women with primary infection, there were two spontaneous fetal losses and one infected baby who had petechiae at birth and unilateral hearing loss at 1-year of age. Information on prevention and hygiene has a positive impact and could significantly reduce the incidence of maternal HCMV infection during pregnancy.
Vauloup-Fellous et al. **(**2009) [51]	*Recruitment and attrition*: From 5312 pregnant women who had unknown immune status or were known to be HCMV seronegative, 127 refused HCMV screening. The infection rate between 12–36 WG was 0.008% per pregnant woman-week, and was significantly lower than the infection rate before 12 WG, which was 0.035% per pregnant woman-week (mid *P* = 0.005). HCMV-seroconversion: 0–12 WG: 0.42% of pregnant women, 12–36 WG: 0.19% of pregnant women (*P* < 0.005). Of the 5 women who seroconverted between 12–36 WG, 2 of the newborns were infected but asymptomatic. Among the 11 mothers with primary infection, there were two spontaneous fetal losses and one infected baby who had petechiae at birth and unilateral hearing loss at 1-year of age. Easy-to-follow information on basic hygienic measures, mainly related to the handling of young children, at the beginning of pregnancy could significantly reduce the incidence of maternal HCMV infection during pregnancy and thus the number of infected fetuses.
Reichman et al. **(**2014) [48]	*Recruitment and attrition*: 56 out of 500 women planning pregnancy, who attended to the fertility clinic, discontinued attending the clinic. Most women were seropositive (79.7%), 16.2% were seronegative and 4.1% (2.7% remote infection and 1.4% primary infection) were found to have evidence of seroconversion at the time of initial screening. Women who were seronegative did not show seroconversion during the year following the start of screening. Cytomegalovirus testing and counselling at preconception seemed effective in reducing HCMV exposure in pregnancy.
Revello et al. **(**2015) [49]	*Recruitment and attrition*: Of the 4096 women in the intervention group assessed for eligibility, 1235 had risk factors. Overall, 745 were enrolled and tested for HCMV antibody and 477 were excluded as IgM-HCMV seropositive. In addition, 13 women declined to participate. Of the 745 women who were enrolled and tested for HCMV IgG and IgM, 343 had IgG +, IgM – and were no further tested. Of the total, 331 women were considered and tested at birth. In the control group, of the 4732 women assessed for eligibility, 1798 had risk factors. Of these, 1265 were excluded (IgG positive, awareness of HCMV, absence of screening for fetal aneuploidy at 11–12 week’s gestation, declined to participate). Of the 553 women who were enrolled and test for HCMV IgG and IgM, 315 were re-tested. *Treatment adherence*: 93% of women reported hygiene recommendations were worth suggesting to all pregnant women at risk for infection. 80% of the women reported substantial or complete compliance with the suggested recommendations. The seroconversion rate in the intervention group (1.2%) was significantly lower than in the control (7.6%) group (Δ = 6.4%, 95% CI 3.2–9.6; *P* < 0.001). 3 newborns with congenital infection were in the intervention group and 8 in the control group (1 with cerebral ultrasound abnormalities at birth). Identification and hygiene counselling of HCMV seronegative pregnant women may represent a responsible and acceptable prevention strategy to reduce primary maternal infection and thus congenital HCMV infection. However, a positive attitude is needed in women as the hygiene recommendations implied substantial and continuous behavioral changes.
Calvert et al. **(**2021) [53]	*Recruitment and attrition*: Of the 3975 of pregnant women, 3097 were not eligible (88.8% not living with a child aged less than four), 13 no blood sample was obtained, 483 were HCMV seropositive and 269 were not recruited. Of the 103 women that agree to participate in the RCT, only 87 participants completed the study. After intervention, knowledge about HCMV was significantly different between participants in the intervention group and participants in the treatment as usual group. Within the treatment group there were significant differences at baseline compared to the 34 WG on how HCMV is transmitted and the possible consequences of congenital CMV for the child. However, in the control group, there were no significant differences in knowledge of how HCMV is transmitted. In addition, women in the intervention group reported less frequent risky activities (kissing children on the lips, eating leftover food) compared to the usual treatment group. No different scores on anxiety and depression were observed between the intervention and treatment as usual groups at baseline or at 34 weeks. Seroconversion in pregnant women in the intervention group was 4.55% and, in the treatment, as usual group was 4.65% Digital antenatal HCMV education is accessible and acceptable to pregnant women and they are willing to adopt behavioral change to reduce their risk of HCMV infection.

*HCMV *Human Cytomegalovirus, *WG *Week´s Gestation, *CI *Confidence Interval, *IgG *Immunoglobulin G, *IgM *Immunoglobulin M, *RCT *Randomized Controlled Trial

Calvert et al., 2021 enrolled 103 pregnant women living with children less than four years old that were randomly divided in the control and the intervention groups. The control group received information through a series of slides about influenza vaccination during pregnancy while intervention group watched a digital educational film with detailed information about HCMV infection and its prevention.

Picone et al., (2009) recruited 3665 seronegative pregnant women during the first trimester visit to the obstetrician. Detailed oral and written information about HCMV infection and its prevention were given to both parents and at around 36 weeks of gestation, a second HCMV serologic test was performed. Following the same procedure for the intervention, Vauloup-Fellous et al., (2009) enrolled 5173 seronegative pregnant women during their first trimester visit to the obstetrician.

Reichman et al., (2014) carried out a retrospective cohort study of 444 women who were attempting pregnancy and were referred to a fertility clinic. Seventy-two seronegative women received detailed preconception counselling about HCMV infection and its preventive measures and every 3–4 months they had a follow-up HCMV serology test.

Finally, Revello et al., (2015) included 646 pregnant women with a control group integrated by women enrolled at delivery who were not informed about HCMV infection, while the intervention group received information about hygiene measures and were prospectively tested for HCMV infection until delivery. Furthermore, in this study authors carried out a reinforcement strategy through sessions during follow-up visits at 18 weeks of gestation and questionnaires every 6 weeks.

### Effectiveness of the interventions on HCMV acquisition

The six selected studies reported the HCMV-specific seroconversion rates as a function of the intervention. Of the three studies with a reported control group [49, 52, 53], only one indicated significantly lower HCMV infection after the intervention (4/331, 1.2%) compared with the control group (24/315, 7.6%) [49]. While no significant reduction of seroconversion was found in Adler et al., (2004) and Calvert et al., (2021) when comparing the control and intervention groups. Regarding the study performed by Adler et al., (2004), a significant reduction in HCMV infection was reported in pregnant women with children younger than 36 months of age who were shedding HCMV (1/17, 5.9%) compared to women with children younger than 36 months of age shedding HCMV attempting pregnancy (10/24, 41.6%). On the other hand, Calvert et al., (2021) reported no significant differences in the seroconversion rate between the end of the first trimester and 34 gestational weeks was 4.55% (2/44) in the intervention group and 4.65% (2/43) in the control group.

The study performed by Picone et al., (2009) reported a reduction in seroconversion after the intervention (5/1951, 0.26%) between 12 and 36 weeks of gestation compared with the first trimester of pregnancy (9/1960, 0.46%). Assuming the nine patients with primary infections had negative serology at 0 weeks of gestation (WG), the count of women without prior HCMV exposure at the start of pregnancy would be the sum of seronegative women at 12 WG (1951) plus nine, totaling 1960.

Similar results were obtained in the study reported by Vauloup-Fellous et al., (2009) in which a significant reduction was also observed after intervention at 12 weeks of gestation (5/2583, 0.19%) when compared with the period before the intervention (11/2594, 0.42%). If we consider that the 11 patients with primary infection (indicated by a low HCMV-G avidity index) had negative serology at 0 weeks of gestation, the number of women without prior HCMV exposure at the beginning of pregnancy would be the total of seronegative women at 12 weeks of gestation (2583) plus 11, amounting to 2594.

Finally, although no comparison was made in the study performed by Reichman et al., (2014), none of the 69 seronegative women who were followed-up until the end of the study seroconverted after receiving counselling at the preconception visits.

### Adherence, changes in behavior and HCMV perception

Most of the studies did not report information related to behavioral changes of perception of HCMV [48, 50, 51, 53] as secondary outcomes. Regarding adherence, it is important to evaluate how well pregnant women follow recommended preventive measures as advised by healthcare providers. Two studies provided information regarding adherence to treatment [49, 52]. In Adler et al., (2004), authors reported no significant differences in adherence to the intervention between the groups of participants (infected and uninfected women and pregnant and attempting pregnancy women). On the other study, 745/932 (80%) of respondents women described following the recommendations often 492/745 (66%) or always 253/745 (14%) during pregnancy being the lack of time the major cause to reduce adherence to the prevention measures [49].

## Discussion

Despite the great Public Health impact caused by HCMV congenital infections as a leading cause of stillbirth, neurodevelopmental problems and hearing loss worldwide, there are no vaccines or therapies commercially available to prevent the infection [19, 33–35]. With this regard, the implementation of hygienic measures in the population at risk stands as the cornerstone to prevent HCMV transmission from the mother to the fetus during pregnancy.

In summary, the findings from this systematic review indicate that incorporating hygiene-focused interventions during pregnancy can to some degree reduce the likelihood of acquiring congenital HCMV infection. Nevertheless, the review highlights a scarcity of studies on preventive measures, and the existing ones vary significantly in terms of target populations, assessed outcomes, and the nature and conditions of implemented interventions. This heterogeneity poses challenges in drawing conclusive insights from the available evidence.

The prevalence of cHCMV infection varies from 0.2 to 2% (average 0.65%) depending on maternal seroprevalence [54]. However, this data come mainly from studies performed in developed regions such as Europe, the USA, and Japan. In low income countries the cHCMV prevalence is higher varying from 6 to 14% [55–59]. The 6 studies included in this systematic review were carried out in five developed countries, and results may therefore not be applicable to other countries with higher prevalence rates. Our results highlight the urgent need to conduct new studies implementing preventive measures in the population at higher risk of infection and transmission. Furthermore, in developing countries in addition to the higher prevalence rates, promoting and implementing hygiene-based measures may be more difficult based on lower socio-economic conditions. In fact, it has been reported that higher educational and social levels are associated with improved patient possibilities to change health behaviours [60, 61].

Regarding the HCMV transmission, in three out of the six studies, seroconversion rates were significantly lower either in the intervention group [49] or after the implementation of hygiene-based measures [50, 51]. It is important to mention that, similarly to previously reported results, the three studies with a significant reduction in HCMV transmission rate were conducted exclusively in pregnant women, [43]. Pregnancy has been commonly defined as a ‘teachable moment” since women are more motivated to improve both the lifestyle and healthy habits compared with non-pregnant women [60, 62, 63]. The response based on emotions during early pregnancy leading to concerns about the fetus health together with the new social role of becoming a mother can motivate pregnant women to modify their lifestyle habits [64]. Based on pregnant women’s interest in maintaining healthy behaviours in this period, implementing protocols to improve the knowledge regarding the HCMV transmission will be ideal and will also increase available evidence of the effectiveness of the preventive intervention. More information is required regarding the following aspects: moderators and mediators of the prevention treatment response, contents, format and adherence to the preventive measures.

In addition to the significant reduction in the seroconversion rate observed after our analysis, the extrapolation of results may not be possible due to the limited number of RCTs, the small sample size and the heterogeneity of the sample. Thus, as previously stated, our results highlight the urgent need to carry out new RCTs involving pregnant women from different socio-economic backgrounds. In addition, except for one study [48], the interventions were based on informing patients of preventive measures instead of counselling and active behavior-changing interventions which have already proven to be effective promoting heathy habits during pregnancy for other pathologies [65–67]. Additionally, counselling is a complex process and the effectiveness of the intervention may be dependent on the specific training and experience of the provider. In the six selected studies, interventions were primarily administered by healthcare professionals such as nurses, midwives, and gynaecologists, potentially lacking specialized training for HCMV infection. Furthermore, these studies lacked clear delineation of the specific interventions, often providing imprecise descriptions, thereby complicating the drawing of definitive conclusions. Consequently, the involvement of professionals specialized in behavior modification could prove instrumental in crafting effective health prevention strategies, proposing and implementing tailored counselling plans during pregnancy.

### Limitations

Some limitations must be considered to interpret our results correctly. (i) Our results may be biased since studies were carried out in high-income regions which make difficult the extrapolation of the results to developing countries. (ii) The number of available studies was small and in some studies the sample size was also reduced, leading to limited representativeness [52, 53]. (iii) It was not possible to conduct a meta-analysis due to the clinical and methodological heterogeneity of the included studies.

## Conclusions

The findings presented in this review highlight the limited and low-quality published evidence currently available, limiting the possibility to make recommendations for clinical intervention. There are only six studies that met the criteria, mainly non-RCT, to study interventions aiming to prevent HCMV infection during pregnancy. It is urgent to develop effective and homogeneous interventions, evaluated in high-quality RCTs. The high number of pregnant women developing complications associated with cHCMV infection worldwide and their clinical burden highlights the need that policymakers should seek to promote research efforts in this area, for example, supporting specialized funding calls. This is particularly important due to the healthcare-related costs associated to this infection. In this sense, further efforts should be done to inform and raise awareness in society about HCMV infection during pregnancy, regardless the serostatus of women, because the associated risk cannot be minimized if they are unknown for the population at risk. Furthermore, interventions need to be replicable, based on theory and evidence, and the study of their effectiveness should be assessed in terms of time, contents and format of the intervention.

Implementation of hygienic measures in pregnant women has potential as the cornerstone to prevent HCMV transmission from the mother to the fetus during pregnancy. Nonetheless, due to the lack of evidence related to the small number and low-quality studies carried out to date, it is not possible to indicate its clinical use, and further studies are proposed with the purpose of clarifying the possible benefits.

### Supplementary Information

**Supplementary Material 1.**

## Data Availability

The data used to support the findings of this study are available from the corresponding author upon request.

## Abbreviations

HCMV: Human cytomegalovirus
RCT: Randomized controlled trials
SNHL: Sensorineural hearing loss
cHCMV: Congenital infection
PRISMA: Preferred Reporting Items for Systematic reviews and Meta-Analysis
