# Supplementary material for: Hygiene-based measures for the prevention of cytomegalovirus infection in pregnant women: a systematic review
Source: BMC Pregnancy Childbirth. 2024 Feb 29;24:172. doi: 10.1186/s12884-024-06367-5 (PMC10905865; doi:10.1186/s12884-024-06367-5)
Supplement: Supplementary file 1 — Supplementary Material 1. [file 12884_2024_6367_MOESM1_ESM.docx]

| **Excluded studies** | |
| --- | --- |
| **Study** | **Reasons for exclusion** |
| 1. Prevention strategies for congenital cytomegalovirus infection | Not intervention |
| 1. A Social Marketing Approach to Building a Behavioral Intervention for Congenital Cytomegalovirus | Not intervention |
| 1. Screening, Prevention, and Treatment of Congenital Cytomegalovirus | Not intervention |
| 1. Maternal and fetal cytomegalovirus infection: diagnosis, management, and prevention | Not intervention. |
| 1. Cytomegalovirus Infection in Pregnancy: Prevention, Presentation, Management and Neonatal Outcomes | Not intervention |
| 1. Cytomegalovirus Infection in Pregnancy: Prevention, Presentation, Management and Neonatal Outcomes | Psychological outcomes |
| 1. Experiences of pregnant women and healthcare professionals of participating in a digital antenatal CMV education intervention | Psychological outcomes |
| 1. A Brief Prenatal Intervention of Behavioral Change to Reduce the Risk of Maternal Cytomegalovirus. A Randomized Controlled Trial | Psychological outcomes |
| 1. Educating women about congenital cytomegalovirus: assessment of health education materials through a web-based survey | Non biological outcomes |
| 1. Development and Assessment of a Prenatal Cytomegalovirus (CMV) Educational Survey: Implementation and Impact in a Metropolitan University-Based Clinic | Non biological outcomes |

**Table S1.** Reasons for exclusion of scientific works from the systematic review
